# Pharmacokinetic/Pharmacodynamic Correlation of Cefquinome Against Experimental Catheter-Associated Biofilm Infection Due to *Staphylococcus aureus*

**DOI:** 10.3389/fmicb.2015.01513

**Published:** 2016-01-07

**Authors:** Yu-Feng Zhou, Wei Shi, Yang Yu, Meng-Ting Tao, Yan Q. Xiong, Jian Sun, Ya-Hong Liu

**Affiliations:** ^1^National Risk Assessment Laboratory for Antimicrobial Resistance of Animal Original Bacteria, South China Agricultural UniversityGuangzhou, China; ^2^Laboratory of Veterinary Pharmacology, College of Veterinary Medicine, South China Agricultural UniversityGuangzhou, China; ^3^Los Angeles Biomedical Research Institute, Harbor-UCLA Medical CenterTorrance, CA, USA; ^4^Division of Infectious Diseases, Department of Medicine, David Geffen School of Medicine, University of California at Los AngelesLos Angeles, CA, USA

**Keywords:** biofilms, *Staphylococcus aureus*, PK/PD, cefquinome, catheter-associated infection

## Abstract

Biofilm formations play an important role in *Staphylococcus aureus* pathogenesis and contribute to antibiotic treatment failures in biofilm-associated infections. The aim of this study was to evaluate the pharmacokinetic/pharmacodynamic (PK/PD) profiles of cefquinome against an experimental catheter-related biofilm model due to *S. aureus*, including three clinical isolates and one non-clinical isolate. The minimal inhibitory concentration (MIC), minimal biofilm inhibitory concentration (MBIC), biofilm bactericidal concentration (BBC), minimal biofilm eradication concentration (MBEC) and biofilm prevention concentration (BPC) and *in vitro* time-kill curves of cefquinome were studied in both planktonic and biofilm cells of study *S. aureus* strains. The *in vivo* post-antibiotic effects (PAEs), PK profiles and efficacy of cefquinome were performed in the catheter-related biofilm infection model in murine. A sigmoid *E*_max_ model was utilized to determine the PK/PD index that best described the dose-response profiles in the model. The MICs and MBICs of cefquinome for the four *S. aureus* strains were 0.5 and 16 μg/mL, respectively. The BBCs (32–64 μg/mL) and MBECs (64–256 μg/mL) of these study strains were much higher than their corresponding BPC values (1–2 μg/mL). Cefquinome showed time-dependent killing both on planktonic and biofilm cells, but produced much shorter PAEs in biofilm infections. The best-correlated PK/PD parameters of cefquinome for planktonic and biofilm cells were the duration of time that the free drug level exceeded the MIC (*f*T > MIC, *R*^2^ = 96.2%) and the MBIC (*f*T > MBIC, *R*^2^ = 94.7%), respectively. In addition, the AUC_24h_/MBIC of cefquinome also significantly correlated with the anti-biofilm outcome in this model (*R*^2^ = 93.1%). The values of AUC_24h_/MBIC for biofilm-static and 1-log_10_-unit biofilm-cidal activity were 22.8 and 35.6 h; respectively. These results indicate that the PK/PD profiles of cefquinome could be used as valuable guidance for effective dosing regimens treating *S. aureus* biofilm-related infections.

## Introduction

Biofilm-related infections are major medical problems and are usually refractory to antibiotic therapy (Costerton et al., 1999). The treatment failures in clinical cases are consistently reported by both clinician and veterinarians (Fabres-Klein et al., 2015). *Staphylococcus aureus* is a pathogen commonly associated with biofilm-related infections such as endocarditis, osteomyelitis, prosthetic joint infections, and catheter-related infections (Parra-Ruiz et al., 2012). Antibiotics that are effective against planktonic bacteria often do not prove satisfactory in eradicating biofilms, as biofilm cells are physiologically distinct from non-adherent and planktonic cells (Widmer et al., 1990). *S. aureus* cells within biofilm are significantly resistant to host defense systems as well as the antimicrobial therapy (Begun et al., 2007). The poor therapeutic outcome may be due to slow bacterial growth rate, limited penetration of the antibiotic and the presence of persister cells (e.g., small-colony variants) within the biofilm matrix (Davies et al., 1998). Therefore, there is a growing need for new approaches to optimize antibiotic regimens *in vivo* for the treatment of biofilm-related infections.

Cefquinome is a fourth generation cephalosporin which used widely in the veterinary industry with antimicrobial activity against a broad spectrum of Gram-positive and -negative bacterial species, and is regarded as highly stable to β-lactamases (CVMP, 1995). The PK/PD profiles of antibiotics could provide an important approach to establish more-effective treatment strategies and to predict the antimicrobial efficacies (Craig, 1998). However, most previous PK/PD studies were focused on planktonic cells and very limited results regarding biofilm infections were reported (Shan et al., 2014). The extrapolation of these results to biofilm cells was problematic for predicting an efficient doing regimen in biofilm-related infections (Blaser et al., 1995). Therefore, in the present study, we evaluated the *in vivo* PK/PD profiles of cefquinome against an experimental catheter-related biofilm infection model in murine due to three clinical *S. aureus* isolates and one non-clinical *S. aureus* isolate.

## Materials and Methods

### Antibiotics and Bacterial Strains

Cefquinome was pharmaceutical grade and purchased from Qilu Animal Health Products Co., Ltd. (Jinan, China). Three veterinary clinical isolates from the endocarditis cases [One methicillin-susceptible *S. aureus* (MSSA; S45) and two methicillin-resistant *S. aureus* (MRSA; M4 and M21)], and one non-clinical isolate (MSSA; F27) were included in the present study. All *S. aureus* strains were identified by MALDI-TOF MS system (Axima-Assurance-Shimadzu). For MRSA isolates, the *16S rRNA* and *mecA* genes were detected using a multiplex PCR assay. MRSA strain ATCC 43300 served as a control.

### *In Vitro* Susceptibility Testing and Biofilm Susceptibility Assay

The minimal inhibitory concentrations (MICs) of cefquinome against planktonic *S. aureus* cells were determined using standard Clinical and Laboratory Standards Institute (CLSI) microdilution method (CLSI, 2008). The minimal biofilm inhibitory concentrations (MBICs), biofilm bactericidal concentrations (BBCs), minimal biofilm eradication concentrations (MBECs), and biofilm prevention concentrations (BPCs) of cefquinome for biofilms were determined using the Calgary Biofilm Device as previously reported (Ceri et al., 1999; Moskowitz et al., 2004; Fernandez-Olmos et al., 2012; Macia et al., 2014; Details in Supplementary Material).

### *In Vitro* Time-Kill Curves

The *in vitro* time-kill curves were determined as previously described (Zhao et al., 2014). In brief, cefquinome was added into MH broth containing approximately 5 × 10^5^ CFU/mL exponentially growing *S. aureus* cells to obtain drug concentrations of 0, 0.5, 1, 2, 4, 8, and 16 × MIC, and incubated at 37°C for 24 h. Samples were removed at 0, 3, 6, 9, 12, and 24 h after incubation and then subjected to 10-fold serial dilutions. Twenty-five microliter of each dilution was then plated onto quadrants of MH agar and incubated at 37°C for 24 h for viable counts enumeration. Results were expressed as log_10_ CFU/mL and the limit of detection was 40 CFU/mL.

### Biofilm Formation Assay

Stationary phase of *S. aureus* cells were resuspended in physiological NaCl solution to an OD_650nm_ of 0.5 (∼10^8^ CFU/mL) and diluted 1:100 into Brain Heart Infusion (BHI) broth supplemented with 0.5% glucose (Seidl et al., 2008). The 96-well plate was inoculated with 200 μL of this suspension and incubated for 18 h at 37°C. Subsequently, the wells were rinsed twice to remove planktonic cells. Biofilm was stained with crystal violet (0.1% in distilled water) for 1 min and washed with PBS three times. After visual observation, the adhering dye was dissolved with 75% alcohol to quantify the biomass measuring optical density at 650 nm.

### Experimental Catheter-Associated Biofilm Infection Model in Murine

The animals used for the *in vivo* experiments were 6-week-old (24–27 g) and pathogen-free female ICR mice (Guangdong Medical Lab Animal Center, Guangzhou, China). Animals were maintained in accordance with the American Association for Accreditation of Laboratory Animal Care criteria. All animal experiments were approved by Animal Research Committees of South China Agricultural University.

The catheter-associated biofilm infection in a murine model was established as previously described (Kadurugamuwa et al., 2003). Briefly, 1-cm segments of 14-gage Teflon intravenous catheter (Abbocath-T; Burns Vet Supply, Vancouver, WA, USA) were infected by study *S. aureus* strains in 3 mL of BHI broth supplemented with 0.5% glucose. After 6–8 h incubation at 37°C, the infected catheters were washed twice with PBS to remove unbound bacteria, and then implanted subcutaneously on each side of each mouse. Our preliminary data demonstrated that the infected catheter contained ∼5 × 10^5^ CFU/catheter (data not shown). At specific time points, tissue fluid (planktonic cells) was aspirated around each catheter segment and plated on MH agar plates for CFU determination. At sacrifice, catheters were removed from the subcutaneous tunnels and rinsed twice with PBS. Subsequently, the catheters were transferred to a separate tube containing 1 ml of sterile PBS. The tubes were placed in an ultrasonic bath (100 W, 40 kHz) and sonicated for 10 min, followed by vortexing for 1 min to remove biofilm cells from the catheter surface. The disaggregated biofilm was then processed to quantify the number of viable cells in the suspension.

### Pharmacokinetics

The animals were administrated intramuscularly using varying doses of cefquinome (2, 8, 16, 32, 64, 128, or 256 mg/kg; six animals/group) as a single administration at 24 h after catheter implantation. Blood samples were collected by retro-orbital puncture following time points: 0, 0.08, 0.17, 0.25, 0.5, 0.75, 1, 2, 3, 4, and 6 h after antibiotic administration. Plasma was immediately isolated by centrifugation at 3000 × g for 10 min at 4°C, and drug concentrations in plasma were determined using a HPLC-ESI-MS/MS method as described previously (Zhou et al., 2015). The limit of quantification (LOQ) and detection were 0.01 and 0.005 μg/mL, respectively. The time-concentration curves of cefquinome were best fitted to a one-compartment model with first-order absorption. PK parameters including half-lives of first-order absorption (T_1/2Ka_) and elimination (T_1/2Kel_), volume of distribution during the terminal phase as a function of bioavailability (*V*_d_/*F*), total area under time-concentration curve (AUC), body clearance as a function of bioavailability (Cl/*F*), the peak plasma concentration (*C*_max_) and the time of maximum concentration (*T*_max_) were conducted using WinNonlin software (version 6.1, Pharsight, St. Louis, MO, USA). The bioavailability (*F*) was calculated as F% = (AUC_i.m._/AUC_i.v._) × 100% (*F* = 98.3%, intravenous PK data not shown). The time courses of multiple administrations in PK/PD analysis were extrapolated from the corresponding single dose PK data obtained in the present study. The protein blinding of cefquinome in mouse was previously reported (7.4%; Wang et al., 2014). In addition, cefquinome PKs in healthy animals were also determined as reference.

### *In Vivo* Efficacy of Cefquinome in Planktonic and Biofilm Bacteria and PAEs

To evaluate the *in vivo* efficacy of cefquinome in the experimental catheter-related biofilm model caused by a representative MRSA strain, M4, animals were treated with a single intramuscular dose of cefquinome (8–256 mg/kg) at 24 h after infection. The control groups received physiological NaCl. At 0, 3, 6, 9, 12, and 24 h post-dosing, animals were sacrificed and tissue fluid around the catheter was collected for planktonic bacterial culture. In addition, the catheter segments were removed aseptically, and sonicated for 10 min as described above, then quantitatively cultured. MRSA densities were expressed as mean log_10_ CFU/mL and log_10_ CFU/catheter ±SD for in tissue fluid and biofilm; respectively.

The post-antibiotic effect (PAE) was calculated with the equation: *PAE* = *T* – *C*, where *T* is the time for the mean growth of 1 log_10_ CFU in planktonic or biofilm bacteria of treated mice after free drug levels in plasma fell below the MIC or MBIC, and *C* is the corresponding time for the untreated control mice (Spivey, 1992).

### PD Parameter Determination in Catheter-Associated Biofilm Infection Model

To evaluate the regimens of cefquinome, including dose levels as well as dosing intervals, in planktonic and biofilm bacteria, the anesthetized mice infected with catheter segments carrying ∼5 × 10^5^ CFU *S. aureus* were treated at 24 h after implantation with single or multiple intramuscular administration of cefquinome. Treatment regimens included total doses ranging from 2 to 512 mg/kg/day administered using twice-daily and once-daily. An untreated group received physiological NaCl intramuscularly. All groups of mice were sacrificed after 24 h of therapy. The catheter segments were removed and tissue fluid was collected for CFU determination as described above.

### PK/PD Modeling and Data Analysis

For PK/PD integration of cefquinome in planktonic and biofilm bacteria, the surrogate indices the duration of time that free drug level exceed the MIC or MBIC (T > MIC or MBIC), C_max_/MIC or MBIC and AUC from 0 to 24 h (AUC_24h_)/MIC or MBIC, were calculated for each animal. The numbers of bacteria in planktonic cells and biofilms were correlated to these PK/PD indices for each of the dosing regimens studied. The *in vivo* PK/PD analysis was performed using the inhibitory sigmoid dose-effect model derived from the following formula: *E* = *E*_0_ + *E*_max_ × *C*_e_^N^/(*EC*_50i_^N^ + *C*_e_^N^), where *E*_0_ is the change in log_10_ CFU/mL of untreated controls (absence of drug), *E*_max_ is the maximal antibacterial effect determined as the difference in log_10_ CFU/mL, *EC*_50_ is the value of the target PK/PD index required to achieve 50% of *E*_max_, *C*_e_ is the target PK/PD indices (T > MIC or MBIC, *C*_max_/MIC or MBIC and AUC_24h_/MIC or MBIC), and *N* is the Hill coefficient that described the slope of dose effect curve (Aliabadi et al., 2003). The correlation between the efficacy and each of these PK/PD indices was calculated using the non-linear WinNonlin regression program (version 6.1, Pharsight; Gebru et al., 2009). *R*^2^ was used to estimate the variance of regression with each of the PK/PD parameters.

To further compare the antibacterial efficacy of cefquinome between planktonic and biofilm cells, the sigmoid dose-response model derived from the Hill equation also was used to calculate the target values of cefquinome that produced the bacteriostatic action, 0.5-log_10_-unit and 1-log_10_-unit of the net bactericidal effect over 24 h (biofilm-static, 0.5-log_10_-unit and 1-log_10_-unit biofilm-cidal values, respectively; Zhang et al., 2014).

## Results

### *In Vitro* Susceptibility Testing and Biofilm Susceptibility Assays

The biofilm susceptibility of the four *S. aureus* isolates is shown in **Table 1**. The MICs and BPCs of cefquinome for the four strains used in this study were nearly identical (0.5 and 1–2 μg/mL, respectively), indicating that cefquinome has high antibacterial activity *in vitro* against planktonic *S. aureus* cells and potential ability on preventing of early biofilm formations. The MBICs (16 μg/mL), BBCs (32–64 μg/mL) and MBECs (64–256 μg/mL) of cefquinome against the study *S. aureus* strain cells within biofilms were significantly higher than their corresponding MIC values. None of these parameters correlated with the methicillin-resistance status of these strains.

**Table 1:** *In vitro* susceptibility testing and biofilm susceptibility assays of cefquinome vs. *Staphylococcus aureus* isolates used in this study.

*S. aureus* Strains	Cefquinome (μg/mL)					Oxacillin	*mecA*
	MIC^§^	MBIC^†^	BBC	MBEC	BPC	MIC	
S45 (MSSA)	0.5	16	64	256	2	0.125	ND
F27 (MSSA)	0.5	16	32	64	1	0.125	ND
M4 (MRSA)	0.5	16	64	128	2	16	+
M21 (MRSA)	0.5	16	32	64	1	16	+

*ND, not determined. §MIC, minimal inhibitory concentration of cefquinome for planktonic cells; ^†^MBIC, minimal biofilm inhibitory concentration, defined as the lowest concentration of drug that inhibited visible biofilm growth in the recovery medium (Moskowitz et al., 2004); BBC, biofilm bactericidal concentration, defined as the lowest concentration that killed 99.9% of the biofilm cells (Fernandez-Olmos et al., 2012); MBEC, minimal biofilm eradication concentration, defined as the minimal concentration of antibiotic required to eradicate the biofilm (0 CFU/peg on plate counts; Ceri et al., 1999); BPC, biofilm prevention concentration, determined using a modification of MBIC assay in which bacterial inoculation and drug exposure occur simultaneously (Fernandez-Olmos et al., 2012)*.

### *In Vitro* Time-Kill Curves

**FIGURE 1:**
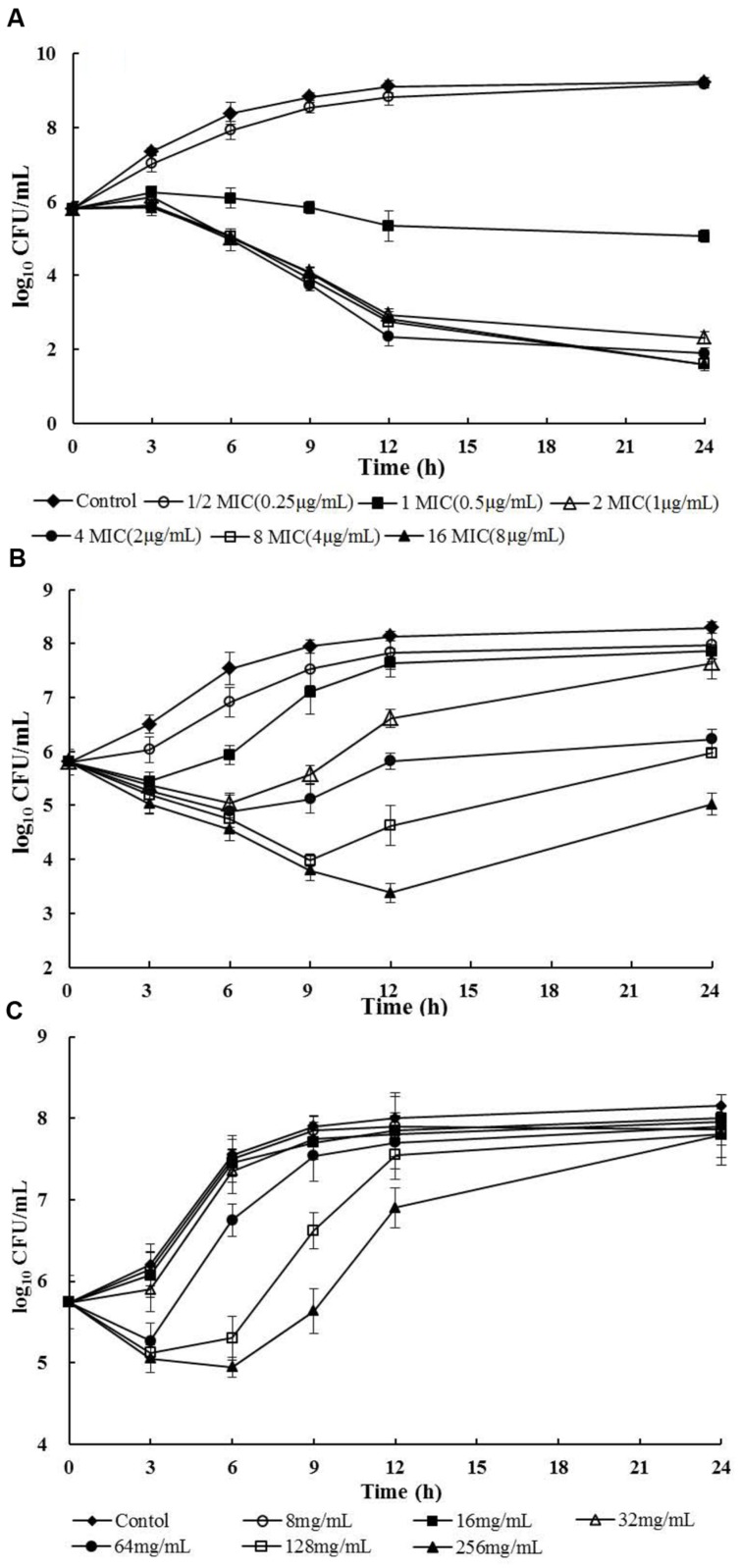
***In vitro* time-kill curves and *in vivo* treatment efficacies of cefquinome against planktonic and biofilm *MRSA-M4* cells in a catheter-associated infection model after a single intramuscular dose of cefquinome (MIC = 0.5 μg/mL). (A)** Cefquinome versus planktonic bacteria *in vitro*; **(B)** Cefquinome versus planktonic bacteria *in vivo*; **(C)** Cefquinome versus biofilm bacteria *in vivo*.

The *in vitro* time-kill curves of cefquinome against planktonic MRSA-M4 were illustrated in **Figure 1A**. *In vitro* killing profiles demonstrated a time- and concentration-dependent feature. Persisting inhibition of planktonic bacterial growth was observed when *S. aureus* was exposed to cefquinome at a concentration of 0.5 μg/mL. At 2 × MIC and all higher concentrations of cefquinome, either bactericidal effect or elimination of *S. aureus* (3- or 4-log_10_-units reduction) was observed during 12–24 h of incubation, while less than 12 h of incubation was insufficient to eliminate all bacteria.

### *In Vitro* Biofilm Formation Assays

**FIGURE 2:**
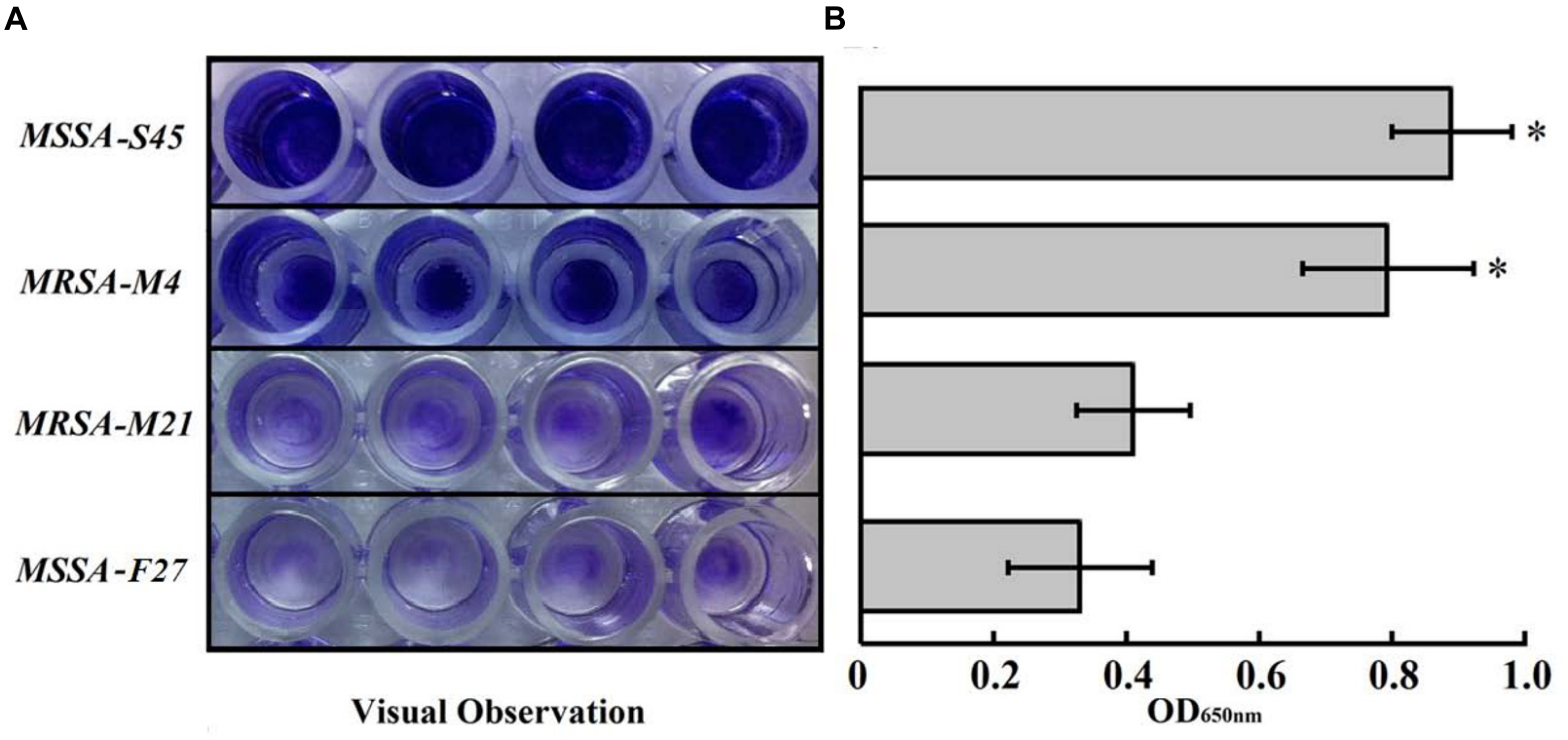
**(A)** Biofilm formation assays of *S. aureus* strains. Performed biofilm was stained with 0.1% crystal violet. **(B)** Quantification (OD_650nm_) of *S. aureus* biofilm formations. Results represent the mean of three independent experiments. Error bars indicate the standard deviation. ∗*P* < 0.01 for S45 and M4 strains versus M21 or F21 strains.

All study strains possessed the ability to form biofilms. Interestingly, MRSA-M4 and MSSA-S45 strains formed significantly greater biofilms as compared to MRSA-M21 or MSSA-F21 strain (*P* < 0.01; **Figure 2**). However, based on the OD_650nm_ values (OD > 2 × OD of media control; 0.2), the MRSA-M21 and MSSA-F21 strains were considered as the strong biofilm producers (Jin et al., 2006). No significant difference was observed between MRSA-M4 and MSSA-S45 biofilms.

### Pharmacokinetics of Cefquinome

The PK parameters of cefquinome in the representative *S. aureus* strain M4 infected mice after a single intramuscular dose of 2–256 mg/kg are shown in **Table 2**. The drug was absorbed and eliminated according to a one-compartmental model with first-order absorption (Supplementary Figure S1). A dose dependency was observed for *C*_max_ and AUC values of cefquinome with ranges of from 3.02 to 287.6 μg/mL and 1.79 to 331.1 μg × h/mL, respectively. The *T*_max_ varied from 0.10 to 0.19 h with a mean of 0.14 h. The elimination half-life (T_1/2Kel_) of cefquinome ranged from 0.22 to 0.48 h. We also demonstrated that there were no significant cefquinome PK differences between healthy and infected animals with different *S. aureus* strains (PK data in healthy animals not shown).

**Table 2:** PK parameters of cefquinome after a single intramuscular administration in catheter-associated biofilm infection model of mice.

Cefquinome (mg/kg)	T_1/2*Kel*_^†^ (h)	T_1/2*K*a_ (h)	*T*_max_ (h)	*C*_*max*_ (μg/mL)	AUC (μg/mL) × h	*V*_d_/*F* (L/kg)	Cl/F (L/kg × h)
2	0.33	0.02	0.10	3.02	1.79	0.54	1.12
8	0.31	0.04	0.15	10.1	6.25	0.57	1.28
16	0.22	0.06	0.16	22.2	11.8	0.43	1.36
32	0.35	0.03	0.12	35.6	22.7	0.71	1.41
64	0.30	0.03	0.11	67.2	37.9	0.74	1.69
128	0.47	0.04	0.17	178.5	165.1	0.57	0.78
256	0.48	0.05	0.19	287.6	331.1	0.73	0.75
Mean ± SD	0.38 ± 0.14	0.04 ± 0.01	0.14 ± 0.03	–	–	0.61 ± 0.11	1.20 ± 0.32

*^†^T_1/2Kel_, elimination half-life; T_1/2Ka_, absorption half-life; *C*_max_, peak plasma concentration; *T*_max_, time of maximum concentration; AUC, total area under the time-concentration curve; *V*_d_/*F*, volume of distribution during terminal phase as a function of bioavailability; Cl/F, body clearance as a function of bioavailability*.

### Antimicrobial Efficacy of Cefquinome and PAEs *In Vivo*

As expected, the *in vivo* activity of cefquinome exhibited time-dependent features both on planktonic cells and on biofilm formations. However, a higher cefquinome administration was acquired to suppress the regrowth of biofilms. The dosage regimen of cefquinome at 256 mg/kg inhibited the planktonic cells for 24 h, but regrowth was observed in the biofilm infections at the same time point (**Figures 1B,C**). Importantly, an approximately 3-log_10_ CFU reduction occurred for planktonic cells, while about 1.5 log_10_ CFU increased in the biofilm infection following 12 h treatment with cefquinome at 256 mg/kg (**Figures 1B,C**).

A positive relationship between cefquinome dosing and PAEs was seen in planktonic *S. aureus* cells (*R*^2^ = 94.9%). However, less than 1 h PAE with the dose range of cefquinome (8–256 mg/kg) was demonstrated for the biofilm cells (**Table 3**). Therefore, significant longer PAE of planktonic *S. aureus* cells was observed as compared to bacteria cells within biofilms (*P* < 0.01; **Table 3**).

**Table 3:** PAE durations of cefquinome against MRSA-M4 after a single dose of administration in catheter-associated biofilm infection model of mice.

Cefquinome (mg/kg)	T > MIC (h)	T > MBIC(h)	PAE duration (h)	
			Planktonic^†^	Biofilm∗


8	1.9	–	0.6	–


16	3.1	0.28	0.9	-0.5


32	4.8	0.47	1.8	-0.2


64	6.0	0.75	2.3	0.6


128	8.7	3.16	3.8	0.3


256	11.5	4.38	5.3	0.7



^†^A positive relationship between cefquinome dosing and PAEs in planktonic cells (*R*^2^ = 94.9%); *P < 0.01 for PAEs of MRSA biofilm versus planktonic cells.

### PK/PD Parameters Integration and Determination

Integration of PK/PD indices of cefquinome with planktonic and biofilm bacteria are showed in **Table 4**. The PK/PD parameters of planktonic cells were significantly greater as compared with the PK/PD profiles of biofilm when single doses of cefquinome at 2–256 mg/kg were administrated (*P* < 0.05; **Table 4**). For instance, T > MICs were ranged from 1.2 to 11.5 h, but the T > MBICs were from 0.28 to 4.38 h. In addition, *C*_max_/MICs and *C*_max_/MBICs were from 6.1 to 575.1 and 0.19 to 17.9; respectively. More importantly, the AUC/MICs were significantly larger than AUC/MBICs (3.6–662 h vs. 0.11–20.7 h).

**Table 4:** Integration of PK/PD indices of cefquinome against planktonic and biofilm bacteria in *S. aureus* catheter-associated biofilm infection model of mice (MRSA-M4).

Cefquinome (mg/kg)	Planktonic			Biofilm∗		
	T > MIC	*C*_max_/MIC	AUC/MIC	T > MBIC	*C*_max_/MBIC	AUC/MBIC
2	1.2	6.1	3.6	–	0.19	0.11
8	1.9	20.2	12.5	–	0.63	0.39
16	3.1	44.5	23.6	0.28	1.39	0.74
32	4.8	71.1	45.4	0.47	2.22	1.42
64	6.0	134.3	75.8	0.75	4.20	2.37
128	8.7	357.1	330.3	3.16	11.2	10.3
256	11.5	575.1	662.2	4.38	17.9	20.7

*∗*P* < 0.05 for PK/PD indices of biofilm versus planktonic cells*.

**FIGURE 3:**
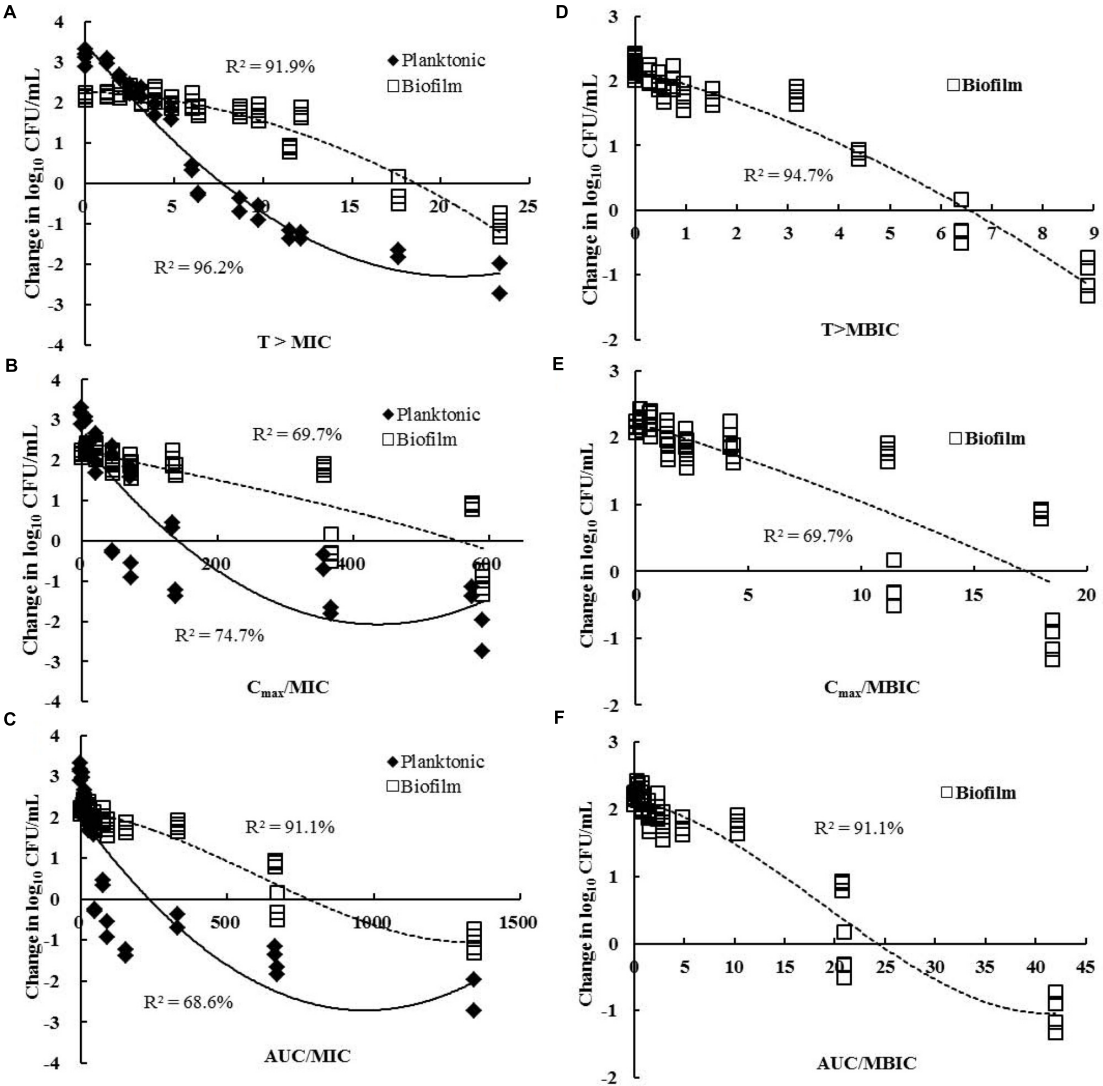
**Relationships between the change in log_10_ number of CFU for *MRSA-M4* and PK/PD parameters of cefquinome**. Each symbol represents the data from each catheter segment or tissue fluid. **(A)** T > MIC; **(B)** *C*_max_/MIC; **(C)** AUC_24h_/MIC; **(D)** T > MBIC; **(E)** *C*_max_/MBIC; **(F)** AUC_24h_/MBIC. The *R*^2^ value is the determination coefficient. Data points below the horizontal line represent killing and points above the horizontal line represent growth. Planktonic cells, filled diamonds; biofilms, and open squares.

The relationship of the change of CFU and the PK/PD parameters of cefquinome between planktonic and biofilm cells was shown in **Figure 3**. T > MIC was the PK/PD index that best correlated with antimicrobial efficacy for planktonic cells (*R*^2^ = 96.2%; **Figure 3A**). However, for biofilm infections, the T > MBIC index (*R*^2^ = 94.7%) showed a better correlation than the T > MIC (*R*^2^ = 91.9%; **Figures 3A,D**). Interestingly, the AUC/MIC parameter exhibited a strong correlation with the *in vivo* efficacy of cefquinome for biofilm cells (*R*^2^ = 91.1%), but a poor correlation for planktonic bacteria (*R*^2^ = 68.6%; **Figure 3C**).

### PK/PD Model Parameter Estimates for the Target Efficacy Against Study Isolates

The PK/PD indices and the corresponding target values of cefquinome required to achieve various efficacies against *S. aureus* M4 in planktonic cells and biofilm infections are listed in **Table 5**. The *EC*_50_ value of cefquinome was 6.61 h for planktonic cells versus 17.4 h for a biofilm infection (*P* < 0.01). The 1-log_10_-unit reduction effect of cefquinome required value of T > MIC at least 10.4 h for planktonic cells, but 22.7 h for biofilm formations (T > MBIC; 8.76 h; **Table 5**). The *in vivo* dose-effect relationship of cefquinome for three additional strains of *S. aureus* in both planktonic and biofilm cells were also calculated using the inhibitory sigmoid *E*_max_ model and a similar result was observed (Supplementary Table S1).

**Table 5:** PK/PD model parameter estimates and target values of cefquinome for T > MIC or MBIC required to achieve the various antibacterial efficacies against a representative MRSA isolates M4 in planktonic or in catheter-associated biofilm infection model.

Parameters and cell type	*E*_max_^†^ (log_10_ CFU/mL)	*E*_0_ (log_10_ CFU/mL)	*EC*_50_ (h)	Static (h)	0.5-log_10_ drop (h)	1.0-log_10_ drop (h)
Planktonic (T > MIC)	–5.64	3.11	6.61	7.25	8.61	10.4
Biofilm (T > MIC)∗∗	–4.40	2.16	17.4	17.6	19.7	22.7
Biofilm (T > MBIC)∗	–3.57	2.13	5.42	6.17	7.35	8.76

^†^E_max_ is the maximal drug effect of cefquinome against biofilm bacteria; E_0_, difference in number of biofilm bacteria (CFU/mL) in untreated group between time 0 and 24 h; EC_50_ is the T > MIC or MBIC value required to achieve 50% of the E_max_; ∗P < 0.05 for T > MBIC in biofilm versus in planktonic cells; **P < 0.01 for T > MIC in biofilm versus in planktonic cells.

In order to predict an effective dose, we determined the dose-response relationships between PK/PD index (AUC_24h_/MBIC) and the *in vivo* efficacy of cefquinome. The dose response curve strongly correlated with the AUC_24h_/MBIC index for all study *S. aureus* biofilm infections (*R*^2^ = 93.1%; Supplementary Figure S2). The target values of cefquinome necessary to produce a biofilm-static action and a 1-log_10_-unit biofilm-cidal action as well as the corresponding AUC_24h_/MBIC index values were listed in **Table 6**. The mean values of AUC_24h_/MBIC associated with stasis and 1-log_10_-unit reduction were 22.8 and 35.6 h; respectively.

**Table 6:** *In vivo* pharmacokinetics and pharmacodynamics model of cefquinome against a *S. aureus* catheter-associated biofilm infection using AUC_24h_/MBIC as the predictive PK-PD index (all study *S. aureus* strains included).

Biofilm PK-PD Parameters (units)	Mean values	*SD*
*E*_max_ (log_10_ CFU/mL)^†^	–3.31	0.17
*E*_0_ (log_10_ CFU/mL)	1.95	0.09
*EC*_50_ (h)	20.3	0.15
Slope (*N*)	3.92	0.79
AUC_24h_/MBIC for biofilm-static action (h)	22.8	1.46
AUC_24h_/MBIC for 1-log_10_-unit biofilm-cidal action (h)	35.6	5.74

*^†^E_max_ is the maximal drug effect of cefquinome against biofilm bacteria; E_0_, difference in number of biofilm bacteria (CFU/mL) in untreated group between time 0 and 24 h; EC_50_ is the AUC_24h_/MBIC value required to achieve 50% of the E_max_; N, slope of AUC_24h_/MBIC-response curve; AUC_24h_/MBIC for biofilm-static action, values which produced E = 0 (no change in bacterial count after 24 h treatment)*.

## Discussion

In this study, an experimental catcher-associated biofilm murine infection model was developed for evaluation of the PK/PD profiles of cefquinome against *S. aureus*, including MSSA and MRSA strains, growing as planktonic cells and within biofilms. To the best of our knowledge, the present study represents the first time PK/PD evaluation of cefquinome *in vivo* in *S. aureus* biofilm-related infections.

Several key insights emerged from this study. First, we investigated *in vivo* PAEs of cefquinome in a catheter-related biofilm infection model due to *S. aureus*, including MSSA and MRSA strains, and observed that the PAEs in biofilms showed significantly shorter than those in planktonic cells (**Table 3**). It is well accepted that the importance of long PAEs for optimizing treatment regimens in clinical practice (Spivey, 1992). Thus, longer PAE is positively correlated with greater *S. aureus* counts reduction in the catheter-related biofilm model which is consistent with previous reports (den Hollander et al., 1998; Ahmad et al., 2015). For instance, a recent study developed in a neutropenic mouse thigh model demonstrated that exposure of planktonic *S. aureus* ATCC 29213 cells to cefquinome led to a visibly longer PAE of 2.9 h as compared to below 1 h PAE observed *in vitro* (Wang et al., 2014). Similarly, another study also proved that the longer *in vitro* PAE could achieve a better *in vivo* treatment efficacy in a rabbit meningitis model (Tauber et al., 1984).

In addition, we demonstrated that cefquinome was fairly effective, causing ∼2- to 3-log_10_ CFU reductions for planktonic cells but only ∼1.0- to 1.5-log_10_ CFU reductions for biofilm cells within the catheter. This observation proved that higher concentrations and longer treatment times of cefquinome are requested to kill *S. aureus* cells within biofilm as compared with planktonic cells in the catheter-associated biofilm infections in murine duo to *S. aureus* strains. Unfortunately, the treatment strategies aimed at eradicating biofilm cells was not achievable in the present model, even at the highest dosing regimen (256 mg/kg; q12h). The lack of the antimicrobial efficacy for biofilm-related infections may be related to intrinsic tolerance of biofilm-grown bacterial populations to antibiotics, as well as the poor penetration of cefquinome through extracellular polymeric matrix of biofilm formations and inadequate antibiotic-exposure for bacterial cells embedded in biofilms (Macia et al., 2014). Other mechanisms such as the contributions of matrix components and chromosomal β-lactamase increases may also lead to this resistance (Hengzhuang et al., 2013).

It is notable that we were able to take advantage of the PK/PD parameters to optimize the *in vivo* efficacy against planktonic cells, as well as biofilm infections in the catheter-related biofilm model. The dose-response profiles of cefquinome for planktonic and biofilm cells showed the best correlation with T > MIC and T > MBIC; respectively (*R*^2^, 96.2% for planktonic cells and 94.7% for biofilms; **Figures 3A,D**). More recently, Wang et al. (2014) investigated the pharmacodynamics of cefquinome for planktonic MRSA infections and also noted that the same index (T > MIC) correlated the best with treatment efficacy in a murine thigh infection model. In our study, although the T > MBIC index exhibited the best correlation, the AUC/MIC of cefquinome was significantly correlated with biofilm killing (*R*^2^ = 91.1%) as compared to planktonic cell infections (*R*^2^ = 68.6%; **Figure 3C**). This was almost equivalent with the correlation of the T > MIC for biofilms (*R*^2^ = 91.9%). Interestingly, the AUC/MBIC index which is based on the antibiotic susceptibility assay of biofilms showed a significant correlation with the *in vivo* efficacy for all study *S. aureus* biofilm cells infections (*R*^2^ = 93.1%). Additionally, considering the higher dosage and longer the treatment periods required to treat biofilm infections *in vivo*, our results suggest that the AUC/MBIC would be the recommended predictive PK/PD index in computation of dosing regimens for *S. aureus* biofilm-related infections.

The development of device-related biofilm infections is correlated with the density of adherent cells on the implant surfaces (Joo and Otto, 2012). Once the bacterial density increases and the colony changes to biofilm formation, the eradication is virtually impossible *in vivo* (Fernandez-Olmos et al., 2012). Therefore, the important treatment strategy for biofilm-producing bacteria is to prevent the progression of early biofilm formation instead of finding a final dosing regimen for eradication of the mature biofilms. In this study, the BPCs of cefquinome for all study *S. aureus* isolates were only slightly higher than their MICs, which is an interesting parameter that could be used with the aim of reducing the cell density to prevent biofilm formation (Macia et al., 2014). More importantly, the present PK/PD model on *S. aureus* biofilms provided an AUC_24h_/MBIC ratio of 35.6 h that could theoretically predict 1-log_10_ CFU biofilm-cidal effect. These results in conjunction with BPC_90_ data of *S. aureus* biofilms may provide an additional approach to the design of dosing regimens that prevent the early stages of biofilms caused by planktonic *S. aureus* cells during antibiotic treatment. Additionally, a recent study also showed that daptomycin and rifampin alone and in combination were successful in preventing *S. aureus* biofilm infections at the early stages in a subcutaneous rat pouch model (Cirioni et al., 2010).

For the treatment of biofilm infections, more frequent administrations are needed to obtain a longer treatment period in form of T > MBIC. However, it is usually impractical to administer drug more frequently in the veterinary clinical trials. Routinely, once-daily or twice-daily schedule is considered a good compliance target. Furthermore, cefquinome (and the majority of β-lactams in general) have short elimination half-lives and the limitations are obvious with this routine dosing strategy (Turnidge, 1998). Thus, new formulations of cefquinome with prolonged half-life profiles should be developed to achieve a better therapeutic outcome in biofilm-related infections.

Our investigations have several limitations. For example, only four representative *S. aureus* strains were evaluated in this study. Thus, the results need to be verified in a larger population of strains. In addition, the combination regimens were not tested against *S. aureus* biofilm infections model. Studies to define the efficacy of antibiotic combinations in the same catheter-related biofilm model are ongoing in our laboratory. Nonetheless, this is the first study to our best knowledge to demonstrate PK/PD relationships of cefquinome against MSSA and MRSA growing as planktonic and biofilm cells in an *in vivo* experimental catcher-associated biofilm infection model. In the present study, cefquinome showed time-dependent activities against *S. aureus* biofilms *in vitro* as well as *in vivo*. In addition, significantly shorter PAEs were observed on biofilms vs. planktonic cells. The PK/PD index of cefquinome that best correlated with anti-biofilm efficacy was the T > MBIC in this study. More importantly, the AUC/MBIC of cefquinome also significantly correlated with therapeutic outcomes for biofilm-related infections. These results could potentially provide a new perspective for establishing appropriate strategies of antibiotic treatment in *S. aureus* biofilm related formation.

## Author Contributions

Y-HL conceived of this study and participated in its design and coordination. Y-FZ designed the experiment and drafted the manuscript. Y-FZ, WS, and YY carried out the *in vivo* animal experiments and *in vitro* time-kill curve studies. M-TT carried out the *in vivo* experiment about the additional non-clinical *S. aureus* strain in the revision of manuscript. YX and JS participated in the data analysis and revision of manuscript. All authors read and approved the final manuscript.

## Conflict of Interest Statement

The authors declare that the research was conducted in the absence of any commercial or financial relationships that could be construed as a potential conflict of interest.
