# Supplementary material for: Pharmacokinetic/Pharmacodynamic Correlation of Cefquinome Against Experimental Catheter-Associated Biofilm Infection Due to Staphylococcus aureus
Source: Front Microbiol. 2016 Jan 7;6:1513. doi: 10.3389/fmicb.2015.01513 (PMC4703793; doi:10.3389/fmicb.2015.01513)
Supplement: Supplementary file 1 [file Image_1.PDF]

Supplementary Material

Pharmacokinetic/Pharmacodynamic Correlation of Cefquinome against Experimental Catheter-associated Biofilm Infection due to *Staphylococcus aureus*

Yu-Feng Zhou, Wei Shi, Yang Yu, Meng-Ting Tao, Yan Q. Xiong, Jian Sun and Ya-Hong Liu*

*** Correspondence:** Ya-Hong Liu; College of Veterinary Medicine, National Reference Laboratory of Veterinary Drug Residues, South China Agricultural University; Wushan Road 483, Guangzhou, 510642, China; E-mail: lyh@scau.edu.cn; Tel: +86 2085287189; Fax: +86 2085284896.

Supplementary Materials and Methods

In vitro biofilm susceptibility assay

Briefly, biofilms were formed on pegs attached to the top lid of a microtiter 96-well plates (Nunc; Thermo Scientific, Roskilde, Denmark). Following biofilm growth, the peg lid was transferred to a new plate containing two-fold diluted antibiotics. After antibiotic incubation, the top lid was rinsed and placed into antibiotic-free media. Biofilms were then transferred by sonication and the viability of biofilms after 24 h incubation was measured either by obtaining plate counts or by reading the optical density at 650 nm (OD_{650nm}).

The MBIC was defined as the lowest concentration of drug that inhibited visible biofilm growth in the recovery medium (Moskowitz et al., 2004). The BBC and MBEC were determined by plating 0.1 mL of each well with no visible growth of microplates used for MBIC determination on Mueller-Hinton agar plates and incubating for 24 h. The BBC was defined as the lowest concentration that reduced the number of colony-forming units (CFU) to 1/1000th of that in the original inoculum (99.9% kill) (Fernandez-Olmos et al., 2012). The MBEC was defined as the minimal concentration of antibiotic required to eradicate the biofilm (0 CFU/peg on plate counts) (Ceri et al., 1999).

The BPC values were determined using a modification of MBIC assay consisting in incubating peg lids with the planktonic inoculum at the time of exposure to different antibiotic concentrations simultaneously (Fernandez-Olmos et al., 2012). The following steps were the same as the MBIC assay described above.

Supplementary Figures

Supplementary Figure 1. Semi-logarithmic plot of mean plasma time-concentration curves of cefquinome in MRSA-M4 infected mice following a single intramuscular injection with doses from 2 to 256 mg/kg. Values are mean ± SD (n = 6).

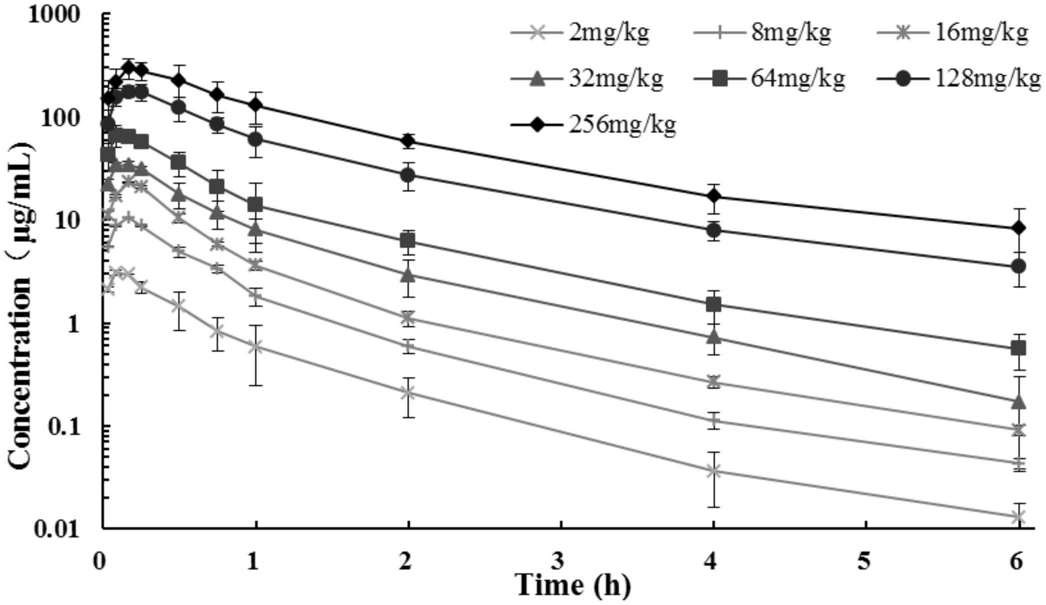

Supplementary Figure 2. The *in vivo* dose-response relationship of cefquinome against a *S. aureus* catheter-associated biofilm infection using AUC_{24h}/MBIC as the predictive PK/PD index (including all study *S. aureus* strains). Each symbol represents the mean and standard deviation from four catheter segments / two mice. The line drawn through the data points was the best fit line based upon sigmoid E_{max} model. R^2 is the determination coefficient.

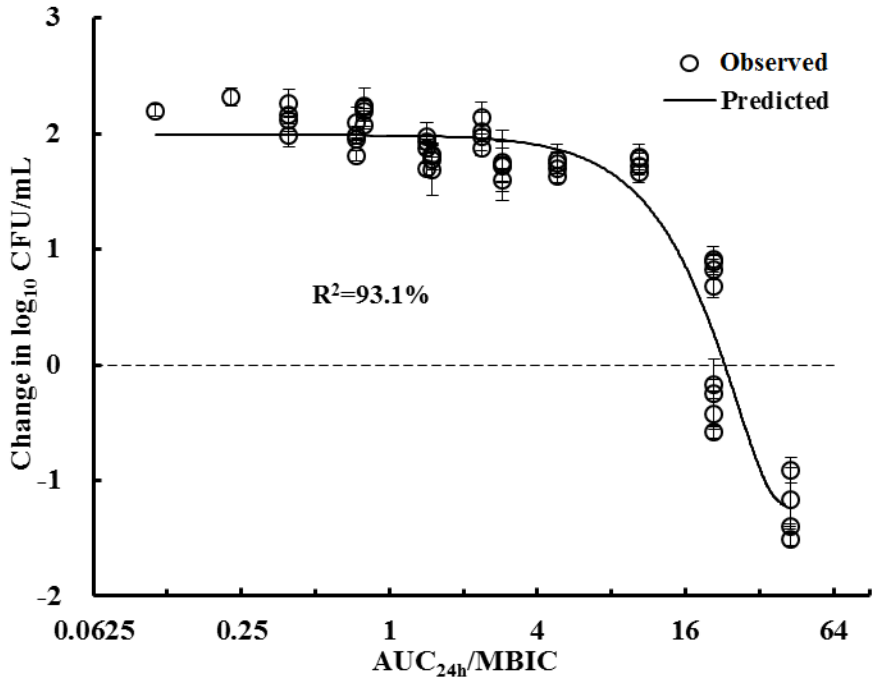

Supplementary Table

Supplementary Table 1. PK-PD model parameter estimates and the target values of cefquinome for T>MIC or MBIC required to achieve various antibacterial efficacies against MRSA-M21, MSSA-S45 and **MSSA-F27** in planktonic or in catheter-associated biofilm infection model.

Parameters and cell type	E_{max} † (log_{10}CFU/mL)	E_0 (log_{10}CFU/mL)	EC_{50} (h)	Static (h)	0.5-log_{10} drop (h)	1.0-log_{10} drop (h)
MRSA – M21						
Planktonic (T>MIC)	-5.81	2.95	7.07	7.31	8.57	10.2
Biofilm (T>MIC)	-4.07	2.06	16.3	16.4	18.3	20.9
Biofilm (T>MBIC)	-3.64	1.97	5.39	5.63	6.45	7.68
MSSA – S45						
Planktonic (T>MIC)	-5.08	3.03	5.93	6.85	8.07	9.81
Biofilm (T>MIC)	-3.87	2.10	16.8	17.6	20.5	24.8
Biofilm (T>MBIC)	-3.07	1.98	5.19	5.98	7.35	8.87
MSSA – F27						
Planktonic (T>MIC)	-5.94	2.93	7.19	7.05	8.31	9.77
Biofilm (T>MIC)	-4.13	1.95	16.4	15.9	17.8	20.2
Biofilm (T>MBIC)	-3.76	1.87	5.29	5.24	6.03	7.12

† E_{max} is the maximal drug effect of cefquinome against biofilm bacteria; E_0, difference in number of biofilm bacteria (CFU/mL) in untreated group between time 0 and 24 h; EC_{50} is the T>MIC or MBIC value required to achieve 50% of the E_{max}.
